# Development of the Home based Virtual Rehabilitation System (HoVRS) to remotely deliver an intense and customized upper extremity training

**DOI:** 10.1186/s12984-020-00789-w

**Published:** 2020-11-23

**Authors:** Qinyin Qiu, Amanda Cronce, Jigna Patel, Gerard G. Fluet, Ashley J. Mont, Alma S. Merians, Sergei V. Adamovich

**Affiliations:** 1 Department of Rehabilitation & Movement Sciences, School of Health Professions, Rutgers Biomedical and Health Sciences, Newark, NJ USA; 2 Department of Biomedical Engineering, New Jersey Institute of Technology, Newark, NJ 70102 USA

**Keywords:** Stroke, Upper extremity, Virtual reality, Telerehabilitation, Serious gaming

## Abstract

**Background:**

After stroke, sustained hand rehabilitation training is required for continuous improvement and maintenance of distal function.

**Methods:**

In this paper, we present a system designed and implemented in our lab: the Home based Virtual Rehabilitation System (HoVRS). Fifteen subjects with chronic stroke were recruited to test the feasibility of the system as well as to refine the design and training protocol to prepare for a future efficacy study. HoVRS was placed in subjects’ homes, and subjects were asked to use the system at least 15 min every weekday for 3 months (12 weeks) with limited technical support and remote clinical monitoring.

**Results:**

All subjects completed the study without any adverse events. Subjects on average spent 13.5 h using the system. Clinical and kinematic data were collected pre and post study in the subject’s home. Subjects demonstrated a mean increase of 5.2 (SEM = 0.69) on the Upper Extremity Fugl-Meyer Assessment (UEFMA). They also demonstrated improvements in six measurements of hand kinematics. In addition, a combination of these kinematic measures was able to predict a substantial portion of the variability in the subjects’ UEFMA score.

**Conclusion:**

Persons with chronic stroke were able to use the system safely and productively with minimal supervision resulting in measurable improvements in upper extremity function.

## Background

Stroke is a leading cause of serious long-term disability in the United States. Projections show that by 2030, an additional 3.4 million people or 3.88% of U.S. adults 18 and older will have had a stroke, a 20.5% increase from 2012 [1]. At 6 months post-stroke, ~ 65% of affected persons continue to have hand deficits that profoundly affect their ability to perform their usual activities [2–4]. Therapy in an inpatient rehabilitation center in the United States only lasts about 2 to 3 weeks, and as outpatients, stroke survivors are typically only seen two to three times a week for short time periods. This volume of intervention falls far short of the volume of rehabilitation needed to re-establish normal hand function. Recently published results of innovative lab-based interventions appear to have a similar problem [5, 6]. Additionally, individuals with disabilities post-stroke have difficulty accessing rehabilitation facilities due to transportation, health and mobility issues. This further reduces training volume. It is therefore imperative to develop an intervention that can be delivered at home, over a period sufficient to elicit improvements.

Although home based systems are increasing in popularity, adherence to unsupervised home exercise regimens is poor across all diagnoses and particularly so in persons with stroke [7, 8]. Low motivation levels are cited as an important barrier [9, 10]. Several small studies have cited higher levels of motivation associated with video game-based rehabilitation activities [11, 12]. Studies observing or measuring the number of repetitions of training activities performed by individuals with stroke, describe subjects participating in video game-like training activities performing more repetitions than subjects performing traditionally presented activities [13–15].

Telemedicine has been broadly defined as the use of telecommunication technologies to provide medical information and services [16]. Recently, innovative telerehabilitation systems have been developed using information and communication technologies to provide rehabilitation services at a distance. Many studies have developed video game driven systems from commercially available gaming consoles such as Wii and Microsoft Kinect [17], however, these systems do not address hand rehabilitation. Other groups, including members of our own team, have examined the use of custom-made telerehabilitation systems [18, 19]. The ideal home based telerehabilitation system must be low cost, easy to setup, and motivating to the user in order to support consistent use. Additionally, it needs to generate progress reports for the user for self-tracking, as well as provide daily monitoring to remote clinicians. Exciting new technologies have now made this approach possible and hold promise for long-term benefit. These technological advances—for the first time—allow for virtual reality games interfaced with discrete finger and hand tracking to be affordable and easy to use.

The Home based Virtual Rehabilitation System (HoVRS) was designed in an attempt to address the above objectives to provide intense upper extremity rehabilitation at home. It allows subjects to access hand/arm rehabilitation without the cost and transportation challenges associated with outpatient rehabilitation. HoVRS consists of four elements: (1) an affordable and commercially available infrared camera specifically designed to capture finger movements—which are not captured by game consoles like Kinect or Wii, (2) multiple engaging games designed to train the hand and arm using commercial gaming mechanics to optimize players’ motivation to perform these activities for long periods of time, (3) monitoring and archiving software that will allow clinicians to design custom rehabilitation interventions, track a patient’s progress, and modify a patient’s rehabilitation program, in-person or remotely, and (4) a secure wireless data connector to collect detailed information on patient movement in real time. The secure communication channel allows for remote monitoring by clinicians, remote technical support, and remote patient and clinician interaction face to face while the patient uses HoVRS. This paper describes the design of HoVRS and presents proof of concept and feasibility data from the first 15 persons with stroke that participated in pilot testing of HoVRS in their homes.

## Home based Virtual Rehabilitation System (HoVRS) design

**Fig. 1:**
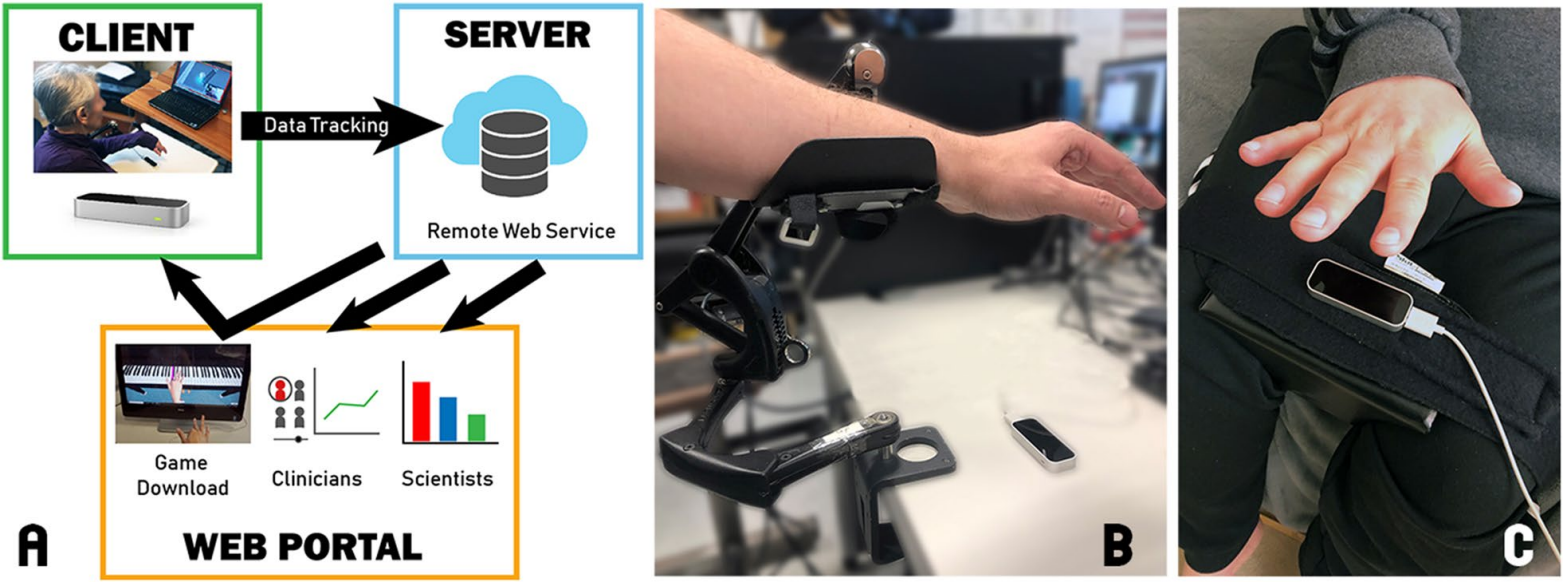
HoVRS sub-systems diagram and types of arm positioning. **a** HoVRS sub-systems diagram: The client-based platform provides hand and arm training. A cloud-based data server provides secure data streaming, analysis and presenting. Therapists can access patients’ progress through web portal. Two different types of arm positioning above LMC: **b** Passive arm support. **c** Hip wedge

HoVRS has two subsystems to deliver home-based training: (1) a patient-based platform to provide the training and (2) a cloud-based online data logging and reporting system (Fig. 1a). In the patient’s home, a cross-platform virtual reality training application runs video games (developed in the Unity 3D game engine using the C# language) on their home computer. The Leap Motion Controller (LMC), a low-cost, commercially available, infrared tracking device is used to capture motion of hand and arm without requiring wearable sensors which may be difficult to put on independently or could potentially restrain movement. This allows the user to interact and control the games with their hand and arm.

### Hardware

#### Motion capture

The LMC consists of three infrared LEDs and two cameras. The functional range of the LMC extends from approximately 25 to 600 mm above the device (1 in. to 2 feet). A data validation study showed the LMC to be accurate and reliable when the target is within its visual area (± 250 mm of the LMC center) [20]. The device’s USB controller reads the sensor data into its own local memory and performs any necessary resolution adjustments. This data is then streamed via USB to the Leap Motion Image Application Programming Interface (API). Using Unity, we programmed the system to feed tracking data into virtual reality activities by calling the Leap Motion API.

#### Hand positioning

Some individuals with severe proximal arm impairment have difficulty maintaining their hand above the optimal LMC capture space for the duration of gameplay. An arm support (Fig. 1b) was supplied to these individuals at the initial visit to provide anti-gravity arm support to assist with positioning their hand above the LMC. This also allows patients that are unable to move their UE through space the ability to transport their hand in the LMC’s workspace. The arm support was positioned at the proximal aspect of the forearm. Therefore, wrist flexion/extension and forearm pronation/supination movements were not obstructed. Several patients demonstrated difficulties producing wrist and forearm movements when moving their upper extremities against gravity without assistance. The addition of the arm support facilitated distal movements in these subjects. Arm supports were mounted on the table where the LMC and laptop were located. If a subject’s living environment did not have the appropriate surface to attach the arm support, we provided an LMC stand to mount the arm support and LMC. The LMC stand was made in-house and was constructed of metal with a heavy and stable base. Another accommodation we provided to enable optimal hand positioning above the LMC was a hip wedge (Fig. 1c), which was secured between a subject’s legs. This was especially helpful for those with some active elbow stability as well as active shoulder rotation movement. The LMC was attached with Velcro on the top of the hip wedge and the subject’s hand was easily placed above the LMC and within its effective range. A third option was a forearm trough. This was used for subjects who only had distal finger movement, allowing them to interact with the system for hand and wrist training. With their arm supported by the trough, subjects were able to focus on finger movement without using the arm and shoulder to stabilize their hand. These accommodations demonstrate the flexibility of the system with regards to physical space and impairment level of the individual.

### Software

#### Games

Rehabilitation games can either be installed by engineers during the initial system setup or downloaded from the HoVRS website at the instruction of an individual’s therapist. After a preliminary configuration session that involves system calibration, subjects start the system for subsequent sessions by choosing a game from a Graphic User Interface (GUI) with a single mouse-click. Games’ initial levels are carried forward from their previous training sessions, eliminating the need for calibration at the start of each session.

Currently, 12 games have been developed which can be grouped into one of four categories: Elbow-Shoulder, Wrist, Hand and Whole Arm (see game descriptions in Additional file 1). In summary, each game trains a specific movement pattern, such as wrist pronation or finger fractionation. The finger games include Car, Bowling, Finger Flying, and Piano. These games utilize the range of whole hand finger flexion/extension calibrations. With the exception of the Piano and Fruit Picking games, these games encourage hand opening to control the speed of movement of a virtual object in order to reach targets and avoid obstacles. The Piano game encourages finger individuation integrated with reaching. Wrist games include Whack a Mole and Wrist Flying and utilize the range of pronation/supination, range of radial/ulnar deviation or range of wrist extension/flexion calibrations. These games encourage the player to practice pronation/supination and extension/flexion in order to successfully catch or hit targets. The third category is shoulder-elbow games which include the Maze, Brick Break, Arm Flying, and Soccer Goalie games. These games require calibrations of vertical and horizontal arm range and use these arm positions to move the object or character in a game. The fourth category is whole arm games which includes Fruit Picking and Fruit Catching. Games in all categories adjust movement of the virtual objects to the range of player movement, calculated based on their calibrations. They all include either multiple levels that increase in difficulty or a single level that adjusts dynamically by algorithms. There is a configuration window that clinicians can use to set up game conditions for variables such as workspace size, activity speed, accuracy demands, etc. Clinicians can also track patient’s performance such as duration of gameplay and game score daily, weekly, and monthly.

#### Algorithms

**Fig. 2:**
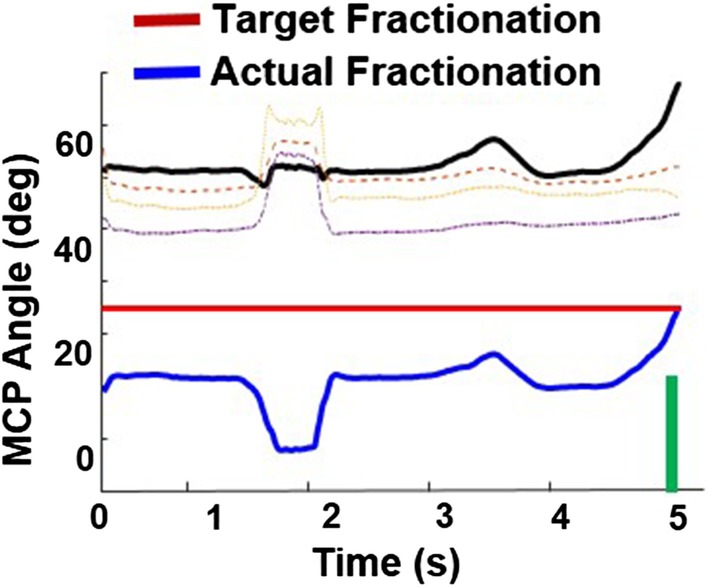
An example of an online algorithm in virtual piano game. An online algorithm is modifying the difficulty levels for the virtual piano game by using the real-time assessment of finger individuation during the piano game (see text for details). The objective for the algorithm is to allow the participants with various levels of finger impairment to successfully press the virtual piano keys while at the same time keeping the activity sufficiently challenging

HoVRS streams kinematic data of the hand and wrist for 22 degrees of freedom in addition to hand orientation and position. These include hand palm position, palm orientation, wrist position, three joint positions from each finger, (metacarpophalangeal joint, proximal interphalangeal joint, distal interphalangeal joint) and fingertip coordinates. These data are used to control the game progression with the help of various online algorithms. There is a target movement that is shaped by each game, for example, the Flying game shapes either finger extension, wrist extension, or shoulder elevation, depending on the specific version of the game. One of the main objectives of these algorithms is to maintain the difficulty levels for each of the games within a prescribed range [21]. Figure 2 shows an example of such an algorithm for the Piano game. The subject was required to flex his active finger, which was randomly assigned by the game, to press the piano key while keeping the other non-active fingers straight. Actual fractionation was calculated as the metacarpal joint (MCP) flexion angle difference between the active finger (solid black line in Fig. 2) and the most flexed non-active finger (dotted line) in real time. A piano key was successfully pressed (green line) when the target fractionation reached the pre-set target fractionation. The algorithm running in the background tracks subject’s successful key press rate. The algorithm increases target individuation if success rate is higher than 80% and decreases it if the rate is lower than 80%. By adjusting the target individuation, the subject is prompted to flex his active finger more if he/she is capable while not frustrating him when he/she is tired.

#### Testing games

The following computer-based tests of hand kinematics were performed along with clinical tests to monitor adaptations to the games played during the pilot study.

1. Hand opening range (HOR): The subject opens their hand as much as possible and closes their hand as tightly as possible. Hand Opening value is calculated as the difference in the average MCP and PIP joint angles across all 4 fingers in these two hand positions. The bigger the HOR value, the better hand opening range.
2. Hand opening accuracy (HOA): The subject controls a cursor that moves up and down by opening and closing their hand. The subject attempts to trace an irregular wave which moves on the screen from left to right at a constant speed. Accuracy is calculated as the root mean square error between the cursor position and the corresponding target point on the wave. Root mean square error is calculated to quantify the differences in cued movement and actual subject movement (smaller error = better performance). The smaller the HOA, the better control of hand opening.
3. Wrist pitch range (WPR): The subject extends and flexes their wrist against gravity with their forearm in a fixed position. Angular difference between these two positions is reported as WPR.
4. Wrist pitch accuracy (WPA): The subject controls a cursor that moves up and down by flexing and extending their wrist. Task and accuracy calculations are similar to HOA.
5. Hand roll range (HRR): The subject moves and holds their hand in pronation and supination with their elbow fixed. Range calculated in a similar fashion to WPR.
6. Hand roll accuracy (HRA): The subject controls a cursor that moves up and down by pronating and supinating their hand. Task and accuracy calculation are similar to HOA.

In addition to the measures described above, the system is capable of collecting other motions, such as radial and ulnar deviation, that we have not yet piloted with stroke subjects. Additional metrics such as speed of hand opening, finger individuation, and smoothness of spatial trajectory of the hand, are available as well making the system capable of monitoring adaptations to the entire library of games offered and customizable to a wide variety of clinical conditions.

## Pilot study methods

### Participants

Subjects were selected for the study based on the following inclusion criteria: (a) 40–80 years old, (b) unilateral right or left sided stroke, (c) score of 22 or greater on the Montreal Cognitive Assessment [22], (d) no hemispatial neglect evidenced by a score of 0 on item 11 of the NIH Stroke scale or severe proprioceptive loss evidenced by the ability to detect a 1-in. passive movement of the fifth digit with eyes closed. (e) Upper Extremity Fugl-Meyer (UEFMA) of 15-58/66, (f) no receptive aphasia, (g) intact cutaneous sensation. Exclusion criteria were (a) orthopedic pathology limiting the ability to perform upper extremity movements without pain (b) other central nervous system pathology.

### Procedure

#### System setup and initial visit

Subjects had no prior exposure to the system before their initial home visit. A Physical Therapist and an Engineer conducted the initial visits. The team evaluated the subject’s home for adequacy of space and furniture for interacting with the system. For subjects requiring antigravity support, the appropriate hardware as described above was provided (see “Hardware" under "Design" section). Finally, we established that the subject’s internet access was sufficient to administer telerehabilitation, transmit and receive daily game data via our Amazon Web Service (AWS) server, and provide later updates to the system.

Once the system was set up in their home, we showed them how to start the games and informed subjects to keep their affected hand at least 4 in. above the LMC. Visual prompts in the games remind them to keep their hand in this range if their hand strays from an optimal position for the infrared cameras to track.

After administering preliminary clinical tests (see below), we performed a series of calibrations that were used to customize the games to accommodate the movement abilities of each subject. These calibrations evaluated active range of motion for hand opening and closing, wrist extension and flexion, radial and ulnar deviation, pronation and supination, shoulder horizontal abduction/adduction, and shoulder flexion/extension. Testing games, outlined above, were then completed for a baseline pre-test.

After the initial calibrations and testing, we showed the subject their first three games starting with one game from each category—Arm, Wrist and Hand. We set the personalized adjustments for each game based on their impairment level, taught them to save and transmit their data to our server, and how to properly store their system.

#### Training program

HoVRS was placed in subjects’ homes for 3 months. A subset of 5 games (Maze, Wrist Flying, Finger Flying, Car, Fruit Catch) out of the 12-game library, at least one from each category (Elbow-Shoulder, Wrist, Hand, Whole Arm) was chosen for this exploratory pilot study. This ensured the sufficient amount of time on each game and standardized the exposure to gaming activities across subjects. We were able to calibrate four of the five games, so all subjects were able to play. The fifth, Fruit Catch, a complex multi joint activity could not be performed by the most impaired subject in the study. Each weekday, subjects were encouraged to play at least 3 rehabilitation activities for a total minimum of 15 min. We did not schedule subjects to use the system at specific times or interact with them during each session in order to examine frequency and duration of subjects’ independent usage.

#### Follow up visits

During the first month, we made visits to their home once a week to check on calibrations, check their game performance, answer any questions, and provide any technical support. In the second month of the study we supplemented 50% of the home visits with video calls to check on their progress, remotely monitor their gameplay, and answer any questions. If they needed technical support, we did it remotely from lab computers. For the final month, we switched entirely to online check-ins and only went for a home visit if they required additional assistance that could not be completed online. During the final visit to remove the system, we repeated the clinical assessments and computer based kinematic tests.

#### Performance data

**Fig. 3:**
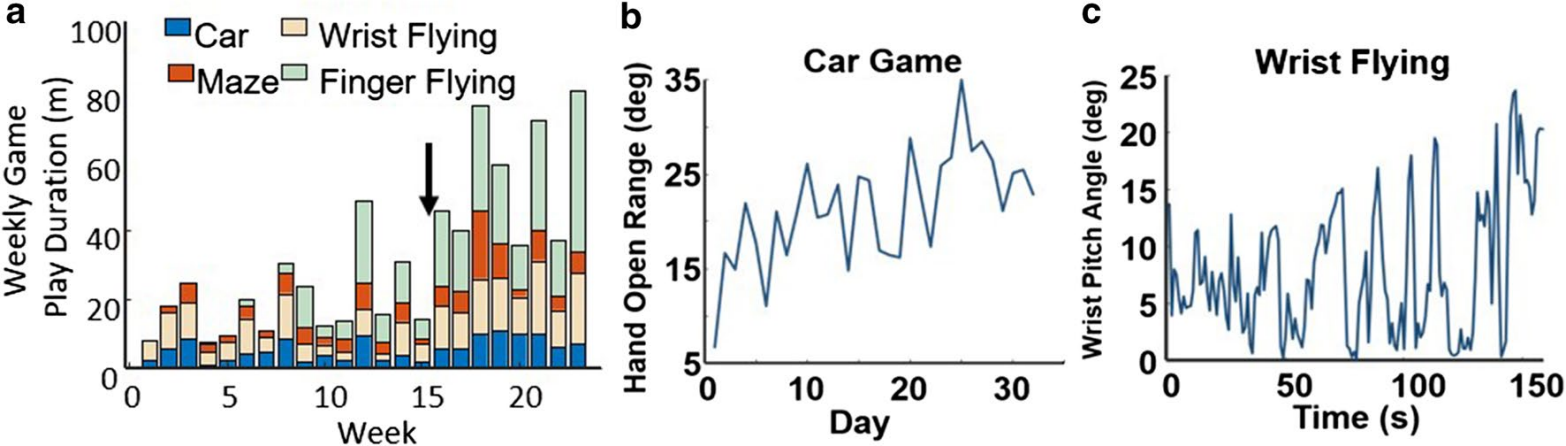
Examples of performance measures captured by HoVRS during training of a representative subject. **a** System weekly usage. The black arrow in panel **a** indicates a new scheme for hand positioning that was introduced by the therapist between week 15 and week 16. That allowed for more successful gameplay, resulting in a substantial increase in adherence. **b** Gradual increase in the range of affected hand opening/closing over the course of training. **c** Modulation of wrist pitch angle during one session of Pitch Flying game

HoVRS logs time and score data as well as finger and arm kinematic data as the subject performs training activities. This provides the clinician supervising the training with the information required to make a wide variety of clinical decisions. For example, Fig. 3a demonstrates weekly training times for all games performed by a representative subject who had the system longer than 3 months. The therapist supervising this subject used these data to determine that he was unable to play the Finger Flying game comfortably on his own. The therapist modified the game by introducing a new positioning scheme, which allowed the subject to use the system more frequently and productively. The effectiveness of clinical decisions can be evaluated using performance data, as demonstrated in Fig. 3b, which shows daily hand opening range of motion over the course of 1 month. This confirms that the calibration and configuration of the Car Game was sufficient to elicit sustained improvement in HOR. Figure 3c demonstrates changes in wrist extension active range of motion during a single session of playing Wrist Flying. This confirms that the subject responded to the game’s calibration and configuration with a steady increase in wrist extension, the game’s target motor behavior. Based on this, the therapist encouraged the subject to train with the system using the current set-up.

#### Outcome measures

The Upper Extremity Fugl-Meyer Assessment (UEFMA) was conducted before and after the 3-month intervention. In addition, six hand and arm kinematics were measured using testing games (see “Design” section above).

#### Statistical analysis

A paired t-test was used to test the difference in UEFMA score between pre and post intervention. Pre and post training test values for kinematic measures and the UEFMA were utilized to examine the relationship between the kinematic measurements provided by LMC and the level of impairment. As there is no gold standard kinematic measure or model of kinematic measures used to describe upper extremity motor function in persons with stroke, we chose an exploratory analysis using Best Subsets Regression analysis to evaluate all 6 kinematic measures and all possible combinations of measures. We identified the models that met the following criteria. (1) the highest R^2^(adjusted). R^2^(adjusted) incorporates the number of predictors in the model to help us choose the correct model. (2) a large R^2^(predicted) that is not substantially smaller the R^2^(adjusted). R^2^(predicted) evaluates a regression model by pulling out each data point and uses the model to predict the missing output, controlling for models with too many predictors. (3) a Mallow’s CP that is not substantially larger than the number of predictors plus 1. Mallow’s CP is a measure of the bias in the sample not due to random sampling error.

## Results

To test the feasibility of this system we included 15 subjects post-stroke. Please see Table 1 for clinical and demographic description of the subjects.

**Table 1:** Clinical and demographic description of the subjects

ID	Age	Gender	Time Since stroke (months)	Hemiplegic side	Initial UEFMA	Final UEFMA	Living situation	Computer skills	Training min/week
S1	67	M	28	Right	40	45	House	Good	46
S2	45	F	192	Right	59	63	Town Home	Basic	88
S3	55	M	204	Right	47	51	Town Home	Basic	168
S4	82	M	84	Right	49	54	House	Basic	100
S5	56	M	36	Right	22	29	House	Advanced	46
S6	57	M	18	Left	56	58	House	Good	58
S7	66	M	30	Left	42	47	House	Advanced	37
S8	62	M	60	Left	40	45	House	Advanced	37
S9	47	M	12	Left	55	56	House	Good	66
S10	50	M	12	Left	15	21	Apartment	Basic	50
S11	35	M	6	Left	30	38	House	Expert	25
S12	63	M	6	Right	46	51	House	Good	134
S13	45	M	6	Left	54	55	Apartment	Basic	70
S14	48	F	84	Right	36	45	Group Home	Good	75
S15	72	M	6	Right	39	50	Basement	Advanced	32

### System support and exercise adherence

All subjects completed the three-month intervention without any adverse events due to the intervention. Patients averaged 47.18 sessions (SD = 17.3) using HoVRS in their homes. The group required an average of 7 in-person support sessions and 5 remote support sessions. They encountered only 6 technical issues that made it impossible for them to perform a session. Four of these issues were resolved remotely and 2 required an in-person visit. Out of 15 subjects in the pilot study, 11 subjects with initial UEFMA range 15–55 used arm support during the first month of training. Subjects were weaned from use of this support as their arm strength increased, only 5 out of 11 subjects needed the arm support for the entire study.

Subjects spent 13.5 h on average using the system. In this study, 4 of 15 subjects (27%) spent more than 15 h on the system which demonstrates > 100% adherence. 7 of 15 subjects (47%) spent more than 12 h on the system which demonstrates > 80% adherence.

### Clinical outcome results

**Fig. 4:**
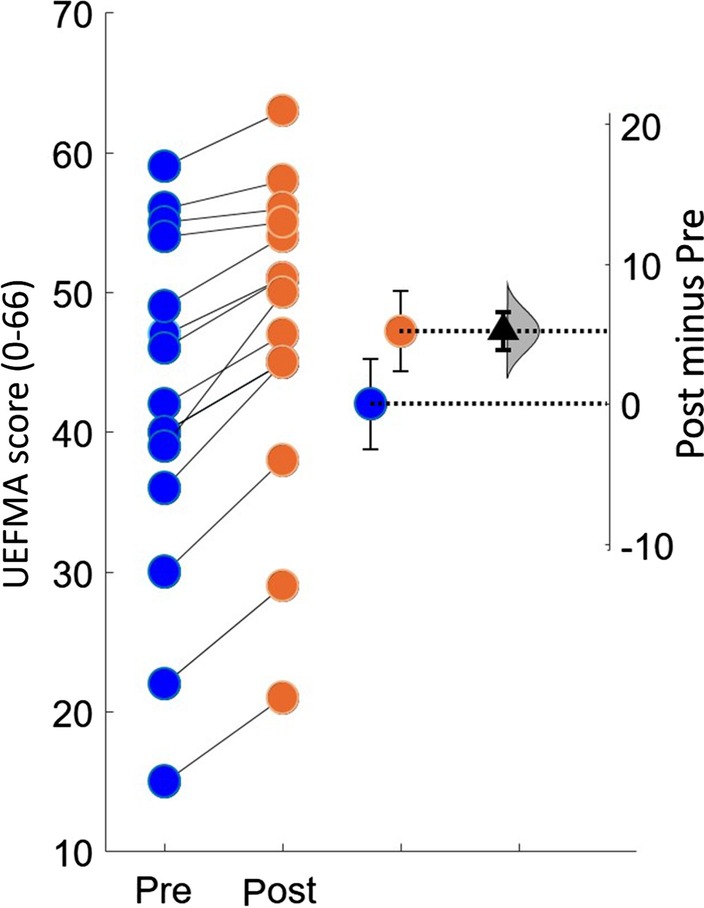
Estimation plots of Fugl-Meyer Assessment. The small circles represent the individual subjects. The blue and orange circles with error bars represent group mean of pre and post UEFMA with their 95% confidence. This is depicted on the axis on the left. The difference between the group means is depicted on the “difference axis” on the right. The 0 point of this axis is based on the group mean of pre UEFMA test. The filled triangle shows the difference between pre and post UEFMA scores. The shaded curve shows the entire distribution of expected sampling error for the difference between the means

The group change average in the UEFMA score from pre to post training was 5.2 (SE = 0.69, p < 0.001, 95% confidence interval [3.66, 6.71]), which exceeds the minimally clinically important difference of 4.25 (Fig. 4) [31].

### Kinematic outcome results

**Fig. 5:**
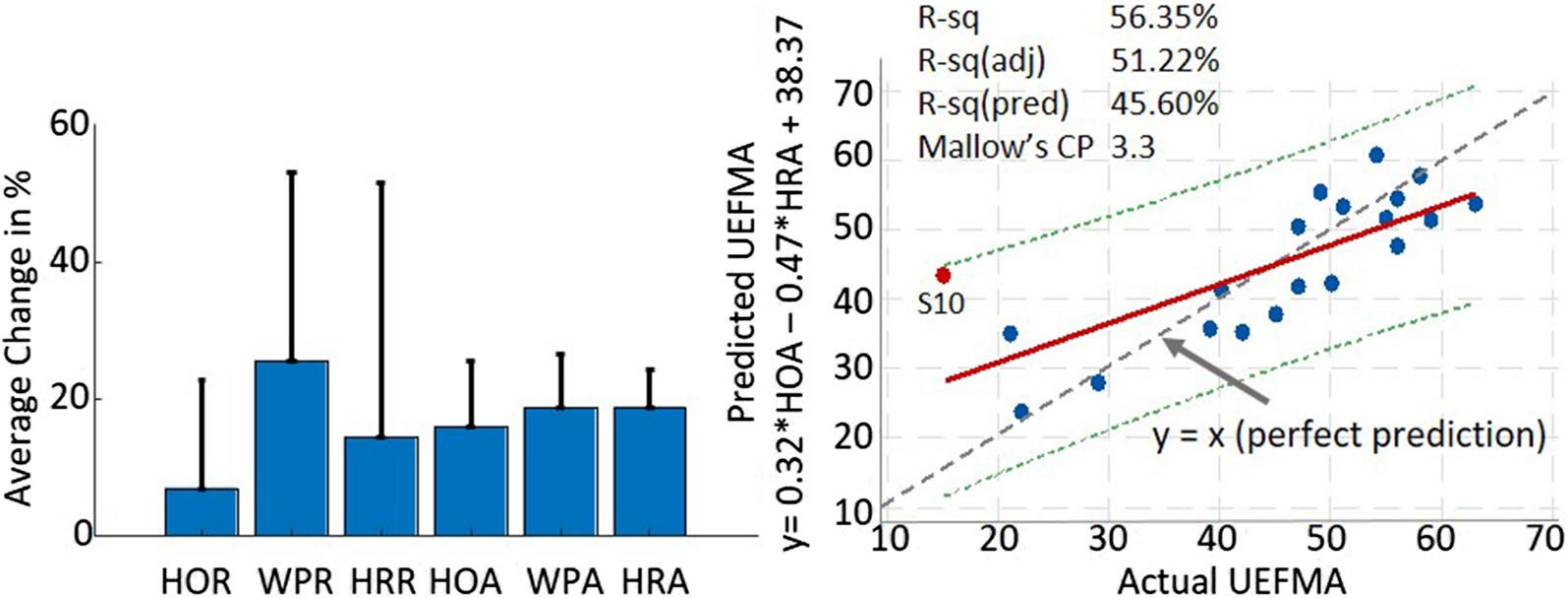
Kinematic outcomes and correlation result. Left panel shows the mean (SD) percentage changes in the range and accuracy of three movements that were used to control the games. After 3 months of intervention, Hand Open, Wrist Pitch and Roll range increased. Accuracy for each movement, which was calculated as the root mean square error, improved. Right panel illustrates the significant relationship between HOA, HOR and UEFMA. Notable is subject S10, the only outlier in this regression model, who also had the lowest baseline UEFMA score in our sample

Since kinematic measures were developed and refined after the feasibility study started, we were only able to pilot the kinematic measures with a subset of the last ten subjects (see "Design"—"Testing games"). All three ROM measures increased: 15.83% for HOR, 27.50% for WPR, and 37.20% for HRR. Subjects demonstrated less error during the tracing tasks (15.76% in HOA, 18.70% in WPA and 18.75% in HRA) as well (Fig. 5 left panel).

### Correlation between kinematics and clinical outcomes

Best Subsets Regression analysis was conducted to understand how hand kinematics measured by HoVRS were associated with the impairment level as measured by the UEFMA. The analysis identified a linear combination of HOR and HRA to have the highest R^2^(adjusted) with acceptable R^2^(predicted) and Mallow’s CP values: UEFMA = 0.32HOR − 0.47HRA + 38.37 (F(2,17) = 10.97, p = 0.001), with an R^2^(adjusted) of 51.2%, R^2^(predicted) of 45.6% and Mallow’s CP of 3.3 (Fig. 5, right panel). The regression analysis identified one outlier (S10, see Fig. 5) who also had the lowest baseline FM score of 15/66 in the group. After excluding the outlier, the best subsets regression analysis identified the following optimal combination of HoVRS kinematic measures to predict UEFMA score: UEFMA = 0.16 * HOR − 0.65 * HRA + 0.09 * HRR + 47.94 ((F(3,17) = 34.45, p < 0.001), R^2^(adjusted) = 85.5%, R^2^(predicted) = 81.3%, and Mallow’s CP = 3.4.

## Discussion

HoVRS was designed as an affordable rehabilitation system that is able to provide stable and reliable hand position, orientation and finger joint kinematic data as well as distinguish various hand shapes, allowing for full hand and finger tracking. Engaging training games and capability of synchronous and asynchronous monitoring from therapists enhance the patients’ adherence. In order to be accessible to more people, HoVRS was designed to be small, easy to transport and fit in a wide variety of homes. People with minimal to no computer skills should be able to use the device.

Overall, full finger and hand tracking was the primary motive for the creation of this system. We investigated other available gesture recognition devices, such as Kai, which reads gesture confidence but does not generate data that is sufficiently robust for meaningful finger tracking. Other tracking devices, such as Kinect and RealSense, do not provide an out of the box finger tracking Software Development Kit (SDK). The LMC provides gesture recognition as well as the raw data for all finger joint positions (ramped gesture values, not just true false) because it has sensors specifically designed to track full hand and forearm movement. In addition to effective tracking, the LMC’s price–performance ratio is strong. Our pilot subjects’ ability to train regularly and independently with the system and the transfer of their training efforts to improved real-world hand and finger function suggest that our goal of full hand and finger tracking was accomplished.

Adherence to technology supported rehabilitation is a complex construct. There were no discernible adherence patterns when considering many of the factors typically posed as possible pitfalls including age, housing situation, level of computer expertise or level of impairment [8]. Technical difficulties can also present a substantial barrier to adherence. The call feature on the HoVRS game menu provided subjects with access to a therapist or engineer via secured video call with a single mouse click. Subjects did not require technical assistance often, but this assistance was available in a timely fashion when it was necessary. Low levels of motivation have a negative impact as well. To augment participants’ drive to train regularly, HoVRS provides real-time positive feedback and encouragement from a virtual therapist. In addition, algorithms running in the background can dynamically change games’ difficulty levels based on subjects’ performance which limits frustration. Our subjects spent about 88 min/week using HoVRS. This result compares favorably to other studies on unsupervised home rehabilitation in stroke [23, 24]. Higher weekly training averages were achieved using other systems [25, 26]. Both of these studies were only 6–7 weeks long, while our study is 12 weeks. Rehabilitation adherence tends to reduce over time, however adherence to HoVRS remained stable for the entire 12 weeks.

A majority of the home-based training systems appropriate for persons with stroke train only proximal UE movements, gross grasp or a single aspect of finger function. This is appropriate in a research setting but becomes less appropriate in a clinical environment. Our pilot group demonstrated a statistically significant improvement in UEFMA score. In addition, 12 of our subjects demonstrated UEFMA improvements that met or exceeded the MCID for chronic stroke subjects [27]. These subjects ranged between 15 and 55 for their pre-test UEFMA score (median 42). We feel that this pattern of consistent, clinically meaningful improvements across our subjects, which included subjects that would not meet the inclusion criteria of many trials of technology supported rehabilitation (at both ends of the impairment spectrum), are a result of the ability of our system to train the shoulder, elbow, wrist and fingers across a variety of movement amplitudes and in three dimensions.

The six kinematic measures piloted, demonstrated improvements in the ability to move in the fashion trained by the games performed during the feasibility study, which establishes an initial argument that these measures might be valid. Interestingly a combination of these kinematic measures was able to predict a substantial portion of the variability in subjects’ UEFMA scores. The two prediction models included measures of hand opening as well as pronation/supination movements. Changes in these movements are both associated with recovery from stroke, in the rehabilitation literature [28]. This suggests that kinematic measures collected by HoVRS by a patient in their home may be able to produce a meaningful measure of a patient’s motor function, when a detailed clinical assessment, performed by a clinician is not possible. This would allow therapists to monitor patients motor control remotely, improving access to therapy and increasing its quality.

## Limitations and future considerations

Limitations of this pilot study include a small sample size and lack of a control group. The current version of HoVRS is not suitable for severely impaired patients (UEFMA < 15) who are not able to move any of their upper extremity joints. Our lab has developed a motorized, admittance controlled hand exoskeleton to help severely impaired patients with participating in gaming activities that require hand opening and closing [29]. Many of the issues requiring in person support were due to difficulties subjects experienced maintaining their hand in the working space of the LMC. Study therapists and engineers have broadened and refined positioning approaches as issues arise. Future development of HoVRS will focus on improving user experience and gameplay, in an attempt to elicit longer and more frequent bouts of training. We plan to allow for a multiplayer mode which has been found to have a positive impact on engagement and adherence by several other groups [30–32]. We also plan to provide additional feedback to subjects’ during training and more detailed performance analysis via the internet for therapists. We will also continue to keep a close eye on new hand tracking devices that come to market as HoVRS is flexible to adapt to any new tracking technology in the future. Future studies will include a more rigorous examination of the kinematic testing battery, a detailed qualitative study of the user experience and a usability study conducted with practicing clinicians.

## Conclusion

HoVRS is able to deliver customized shoulder, elbow, wrist and finger rehabilitation training to subjects in their home setting with minimal in person instruction or assistance that elicits meaningful improvements in hand function.

## Supplementary information

**Additional file 1:** Rehabilitation game descriptions.

## Data Availability

The data sets used and/or analyzed during the current study are available from the corresponding author on reasonable request.
