# Supplementary material for: Development of the Home based Virtual Rehabilitation System (HoVRS) to remotely deliver an intense and customized upper extremity training
Source: J Neuroeng Rehabil. 2020 Nov 23;17:155. doi: 10.1186/s12984-020-00789-w (PMC7685660; doi:10.1186/s12984-020-00789-w)
Supplement: Supplementary file 1 — Additional file 1: Rehabilitation game descriptions. [file 12984_2020_789_MOESM1_ESM.docx]

**Additional file 1: Game Descriptions**

| 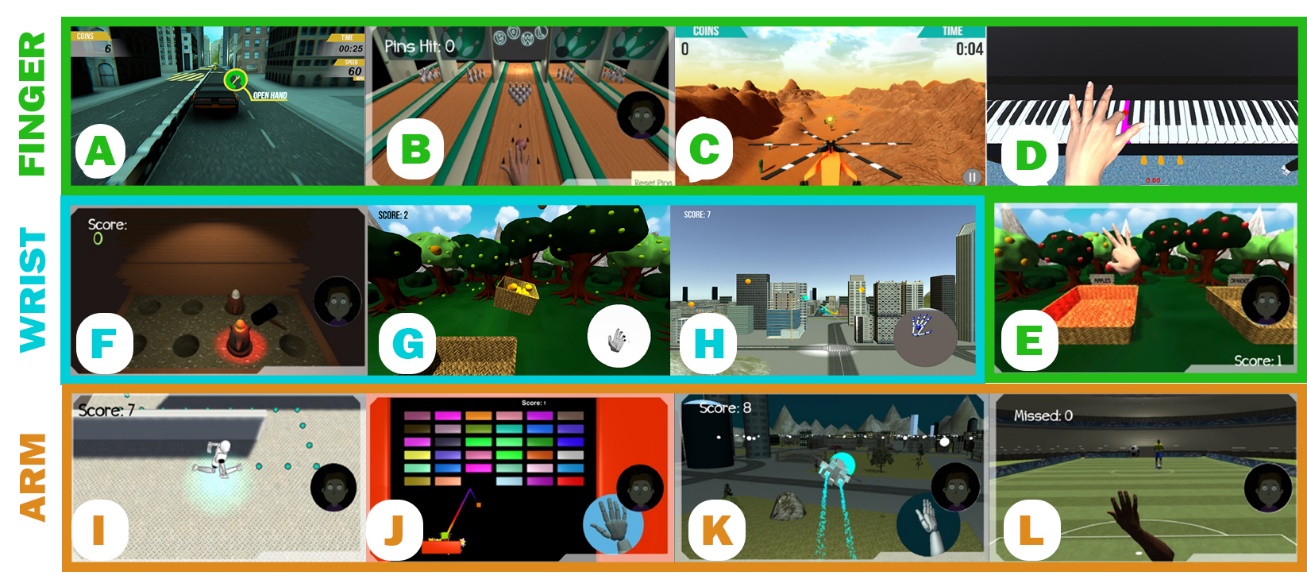 |
| --- |
| Figure S1: Screen shots of 12 rehabilitation games for hand and arm. Finger games include Car, Bowling, Finger Flying and Piano. Wrist games include Whack a Mole and Wrist Flying. Elbow-Shoulder games include the Maze, Brick Break, Arm Flying, and Soccer Goalie games. Whole arm games include Fruit Pick and Fruit Catch. |

- **Car game** (Figure S1A)

Effectors: Fingers, wrist

Calibrations: Range of hand opening, pronation/supination

Set in the streets of a city, the player controls the movement of the car in order to reach the finish line, avoiding obstacles on the road and collecting as many coins as possible for a higher score. The car’s speed correlates to the player’s hand opening. Opening the hand increases the car’s speed and closing the hand slows the car down. Levels increase in difficulty, with added computer competition and obstacles such as speed bumps and traffic cones. In the first level of the game, players practice controlling the speed of the car with their hand opening; the sensitivity of car speed is calculated based upon the player’s range of hand opening calibration. The second level introduces speed bump obstacles and players are required to close their hand to reduce car speed and successfully make it over the bumps. To motivate players to open their hand and speed up the car in between speed bumps, there is a computer-controlled car that players are racing against in addition to the stopwatch that shows their time to complete the level. This competitor car’s speed is calculated using 80% of the player’s maximum calibration values. The third level introduces pronation and supination to change lanes. There are no obstacles on this level so that the player can become acquainted with switching lanes in addition to the original mechanics of controlling the speed of the car. For the following level, there are cones randomly generated in both lanes blocking the path of the car so that the player is required to use pronation and supination to switch lanes, collect the coins, and reach the end of the level. The final level of the game includes a combination of all the obstacles that the player passed to reach this level - speed bumps and traffic cones. Within an adjustment menu, the frequency of obstacles can be adjusted for the player to hold their gestures longer or to increase the rate that they are completing these gestures. Amount of “car health” can also be adjusted to increase the amount of obstacle damage that the car can take before restarting the game level. We generally start with a higher car health and lower object frequency, increasing the number of obstacles as the player increases their performance on the game.

- **Bowling** (Figure S1B)

Effector: Fingers, arm

Calibrations: Range of arm movement along horizontal plane

In a bowling alley, the player is controlling a virtual hand that mimics their hand movement. In order to successfully bowl, they must place their hands within a designated space near the bowling ball which is indicated by a light when they are within the correct distance. To knock over the bowling pins, the player must extend their arm and open their hand to apply force to the ball. We are able to adjust the proximity of bowling pins through a slider on an adjustment menu and move the bowling pins further away as the players are able to increase the amount of arm extension and hand opening.

- **Finger Flying** (Figure S1C)

Effector: Fingers

Calibrations: Range of hand opening

The player is controlling an airplane’s vertical movement as it flies to get through to a landing point in a desert. The plane flies downward when they close their hands to make a fist and then the plane flies upward when they open their hand. As the plane goes further, however, the gasoline levels decrease and the player is required to collect power ups that fill their tank and allow the plane to continue moving. If the gas meter reaches zero, the game ends and the level will restart. There are also stars to collect in the air that allow the player to increase their score. As levels increase and the player flies further through the desert, the powerups and stars are placed at greater distances, so that they are required to open their hand for an extended amount of time to reach them. The final level of this game introduces enemies that players need to avoid crashing into in order to get to their landing zone. In an adjustment menu, we are able to adjust the speed of the plane, the sensitivity of plane movement, and also the amount of power ups and stars that are generated.

- **Virtual Piano** (Figure S1D)

Effector: Fingers

Calibrations:

In this game, the player controls a virtual hand that interacts with a piano, practicing finger individuation to play a song. Before the interaction begins, the game plays a short tune that the player will be replicating, indicating which keys the player will be using as well. Once the piano is finished showing the song, the first key that needs to be pressed lights up and a red sphere indicates which finger needs to be used. The key press will only be successful if there is finger individuation and the amount of which is adjusted in game with an algorithm.

- **Fruit Picking (**Figure S1E)

Effector: Arm, fingers (thumb and index)

Calibrations: Horizontal and vertical arm range

Set in the same environment as the “Fruit Catch” game, the player controls a virtual hand that mimics the movement of their own hand. There are trees with fruit hanging on them and the goal is to pick the fruit and sort them into the correct baskets without dropping them. In order to pick the fruit, the player’s thumb and index finger need to be in proximity of the fruit and they need to make a pinching gesture. Once they complete this gesture, the fruit is magnetized to the area where their fingers are pinching and will follow the finger position until the pinch is released. When the hand is hovering over the correct basket that corresponds to the picked fruit, the basket lights up and they can drop the fruit inside to collect a score. If they drop into the incorrect basket, they lose a point instead.

- **Whack A Mole** (Figure S1F)

Effector: Wrist, arm

Calibrations: range of pronation/supination

Whack A Mole has eight holes that the player needs to hit the moles as they emerge from the holes. The player controls a large hammer that follows their hand position in the horizontal plane and rotates along with their pronation and supination, based upon the amount of pronation and supination that they were able to complete during their initial range calibration. The game will not allow the player to whack the moles by only touching the hammer to them, the game requires the player to supinate and then pronate their hand in order to complete a successful hit. The moles start the game by rising slowly, but increase in speed based on the player’s performance.

- **Fruit Catch** (Figure S1G)

Effector: Arm, wrist

Calibrations: Pronation and supination

Set in a field of fruit trees, the player controls a fruit basket used to collect falling fruits. The fruit basket will move in the horizontal plane following the player’s hand position, but will only move if the player’s hand is supinating, based on 80% of their supination calibration value. The objective of the game is to position the basket below falling fruit to collect as many as possible. If correctly positioned below falling fruit, the fruit basket lights up so that the player knows to keep their hand in this position. They have to hold the supination position while moving the basket horizontally in order to balance the fruit inside and prevent it from rolling out onto the ground. Once collected, the player has to move their hand towards a static collection basket and pronate their hand to rotate the basket to unload the fruit. There is an algorithm involved in this game in order to adjust the speed and frequency of the falling fruit to the player’s performance. When the player catches 80% of the fruit and deposits it into the static basket, the speed of the falling fruit increases; if they continue to miss the falling fruit, the speed and frequency of the spawning will slow down.

- **Wrist Flying** (Figure S1H)

Effector: Wrist

Calibrations: Range of wrist extension/flexion

Flying through a city, the player controls a helicopter with flexion and extension of their wrist. The plane moves vertically - the pitch of the plane rotating with the pitch of the player’s wrist. The speed of the plane is constant and can be set by a slider within the adjustment menu that the therapist can access. While controlling the vertical position of the plane, the player is aiming to collect as many spheres as possible to increase their score. As levels increase, the spheres are placed at greater vertical distances to promote increased wrist extension and flexion and for the player to hold these positions for longer periods of time.

- **Maze** (Figure S1I)

Effector: Arm, fingers (later levels)

Calibrations:

The player controls a virtual robot character through various mazes. The character’s movement is based on the player’s hand position in the horizontal plane of the LMC workspace. Moving the arm forward and away from the body (and from the LMC), for example, will move the character forward. To collect a greater score, the player has to hold their arm and hand position steady to follow a path of successive spheres and collect them. The ultimate goal of the game is to navigate the maze to find the ladder, which will take the character to the next level. The first half of the game involves increasing difficulty with maps increasing in size, navigation past moving walls, and navigation around dead ends. The second half of the game increases difficulty with narrow walkways that the player has to lead the character over without falling off the edge, otherwise the level will restart. Final levels of the game require players to close and then open their hand in order to jump over obstacles. The speed of the running robot is constant and this value can be changed via slider in the adjustments menu.

- **Brick Break** (Figure S1J)

Effector: Arm or wrist

Calibrations: range of radial/ulnar deviation, range of flexion/extension

The player controls the horizontal movement of a paddle in order to control the path of a ball that bounces and breaks the bricks above. This game has two modes of play involving the wrist and arm. In wrist play mode, the player’s finger position, as they practice flexion and extension, controls the position of the paddle. In arm play mode, the player’s hand position as they move their arm left and right in the horizontal plane controls the position of the paddle. The mode of play as well as the scaling of the motion can be changed within the adjustment menu.

- **Arm Flying** (Figure S1K)

Effector: Arm, wrist

Calibrations: Range of arm movement along the horizontal plane

The player is controlling the same helicopter through the city as “Wrist Flying”, however, they are controlling it along the horizontal plane. The plane’s horizontal position is controlled by the player’s distance from the LMC in the x direction. The plane’s banking and turn angle is controlled by the player’s pronation and supination. The objective of the game is to collect as many spheres as possible while they are flying around the city at a fixed vertical position and speed.

- **Soccer Goalie** (Figure S1L)

Effector: Arm

Calibrations:

This game takes place in a soccer field, with the player controlling a virtual hand. They are standing between the goal posts as a goalie, while another computer-controlled player is kicking soccer balls at them. The objective of the game is to block as many balls as possible from getting through to the goal post by waving a virtual hand left and right. The frequency and speed of the soccer balls is based on an algorithm that adjusts with the player’s performance. When the player blocks at least 80% of the incoming soccer balls, the spawn rate will increase. This will also scale down when they are missing more than 20% of the soccer balls.
